# LTP sensitization and clinical features: diagnostic role of microarray tool

**DOI:** 10.1186/2045-7022-1-S1-P5

**Published:** 2011-08-12

**Authors:** Annalisa Ferlisi, Paolo Colombo, Giuseppe Liotta, Angela Bonura, Stefania La Grutta

**Affiliations:** 1 Consiglio Nazionale delle Ricerche, Istituto Biomedicina e Immunologia Molecolare, Palermo, Italy; 2 Agenzia Regionale per la Protezione dell'Ambiente, Palermo, Italy

## Background

The use of the microarray tool is an essential advantage in the allergy diagnosis process in patients with multiple sensitizations showing clinical patterns attributable to the underlying cross-reaction to a single or multiple panallergen. Among panallergens LTP is a thermostable and resistant to pepsin digestion allergen. This features makes it a potent food allergens and explains the frequent development of systemic symptoms.

## Objective

Identify the allergen source which is causing complex clinical patterns in pediatric patients with multiple sensitizations.

## Methods

We studied 11 oupatients children (M 6 F 5, mean age10,45 years, range 6-17 years) with multiple sensitizations and high degree of severity of systemic manifestations: 4 patients had generalized urticaria, 5 patients had angioedema, and 2 patients had anaphylaxis. All children filled a questionnaire related to the personal history of allergy and underwent to the SPT with commercial extracts and fresh food. The in vitro test were performed to assess the total IgE, eosinophil count and to search the specific IgE recombinant allergenic molecules with microarray technique.

## Results

The most significant result is the finding of sensitization to the family of panallergens LTP in 7 of 11 patients. In the majority of patiens (63.6%) with high severity of clinical manifestation, e.i. anaphylaxis and generalized urticaria the LTP sensitization was the only allergen source confirming the role of this molecule in the cross-reaction mechanism.

## Conclusion

This study provided us a good information in the diagnostic evaluation process of pediatric patients with allergic multiple sensitizations and serious clinical patterns in order to plan in real life the long-term management considering the applicability of preventive measure.

